# Hubris and Sciences

**DOI:** 10.12688/f1000research.13848.1

**Published:** 2018-02-01

**Authors:** Eleftherios P Diamandis, Nick Bouras

**Affiliations:** 1 Department of Laboratory Medicine and Pathobiology, University of Toronto, Toronto, Ontario, Canada; 2 King’s College London , Institute of Psychiatry, Psychology and Neuroscience , London, UK

**Keywords:** Leadership, Hubris, Behaviour, Science

## Abstract

There has been an increasing awareness of the importance of leadership and decision making, including scientists and academics, over recent times. By whom and how decisions are made can have serious implications across all levels of society. Several people have been successful in their life and have been inflicted by excessive pride and self-confidence. There are times when the manifestations of such behaviours demonstrate noticeable signs of narcissism and on extreme cases, hubris. Hubris is an old concept originated from the Greek mythology.  The risk of hubris affects politicians, leaders in business, scientists, academia, the military, entertainers, athletes and doctors (among many others). Power, especially absolute and unchecked power, is intoxicating and is manifested behaviourally in a variety of ways, ranging from amplified cognitive functions to lack of inhibition, poor judgment, extreme narcissism, deviant behaviour, and even cruelty. Hubristic behaviour of overconfidence, extreme pride together with an unwillingness to disregard advice makes powerful people in leadership positions to over-reach themselves with negative consequences for themselves and others. As the dangerous consequences of hubristic behaviours become more apparent and well described it is imperative that individuals, organisations and governments act to prevent such phenomena. Responsible leaders, including acclaimed scientists should exercise greater humility to the complexity and inherent uncertainty of their activities and strive to seek out and challenge hubristic behaviours.

## The concept

There will be times when we think that audacity is the route to major discoveries and scientific breakthroughs. How often, however, do we question ourselves if what we know is a real knowledge and not some kind of information? How long will it take for the knowledge that is currently known to become outdated or obsolete? “I learned early in my career the dangers of being too entrenched in what I knew” stated Elisabeth Nabel ^1^, Professor of Medicine at Harvard Medical School, revealing “how limits of knowledge can be a weakness and how ignorance can be strength”. She continued by saying that “none of us in science and medicine have the answers we tell others we have, because the universe of what we don’t know dwarfs what we do know”.

There are times in our life that we must accept that what we thought to be correct will be redefined. For example, stomach ulcer was believed for decades to be related to stress and proved to be a bacterial infection. There are also times we would felt that our pride was hurt, or our confidence was cracked by facts that we had accepted as certain. There is nothing wrong about being wrong. Consequently, humility is essential to be embedded by all of us in everyday life. We should be ready to accept that most of what we know might change over time.

## Humility

Humility is defined by the Oxford dictionary as the “quality of having a modest or low view of one’s importance”. Humility is the element that prevents a sense of overconfidence and exaggerated self-importance and helps to avoid being inflicted by hubris. The Greek philosopher Socrates was proclaiming that “one thing I know is that I do not know anything”, which epitomizes his sense of humility. It is rather surprising that humility is not formally taught at any level of education. Most of us are left to self-discover humility, and many, never discover it. The opposite may happen too. A number of objectively clever, creative and successful people may develop megalomania, which, when left unchecked, can reach the level of hubris.

Humility is necessary in science and technology since the remarkable contemporary advances and discoveries could encourage a feeling of acquired excessive power, with the consequence of developing hubris. Remarkably, some recent discoveries, such as the CRISPR-Cas gene editing technology, may encourage some to “play God” due to their supposed capability to alter the natural abilities of future generations of humans through genomics. In such instances, the discoverers of such technologies forget that they did not really create anything themselves, but they merely understood how microbes developed these tools through millions of years of evolution. The huge financial and commercial pressures affecting scientists in current times, together with those in academia for increased productivity, successful tenders for research grants and high impact factor publications add up to vulnerability of exhibiting behaviour and personality changes or traits manifested as hubris. Many successful people have frequently overestimated their own abilities and believed that their performance is superior to others by imposing excessive pride and self-confidence to themselves. There are times when the manifestations of such behaviours demonstrate evident signs of narcissism and on extreme cases, hubris.

## Hubristic behaviour

The term hubris derives from the Greek mythology, signifying the dangerous combination of over-confidence, over-ambition, arrogance and pride. In the ancient Greek world hubris was considered to be one of the most dangerous traits one could exhibit. In the classical Greek myth when Daedalus and Icarus escaped from the labyrinth in Crete, Daedalus advised his son not to fly too low, in order to avoid being too close to the moisture of the sea or, too high and close to the heat of the sun, as he had used thread and wax to make their wings. Despite this advice, an excessively exuberant Icarus flew too close to the sun and his wax wings melted causing him to fall into the sea. Icarus’ hubris, his disobedience of his father in flying too high, is a cautionary tale about humility and restraint, the danger of audacity.

Hubris has been seen in all walks of life including politics, business, the military, scientists, academia, entertainment, sports and medicine (among many others). In aviation, investigations into fatal plane accidents identified erroneous decisions by the captain whose position of power in the flight deck dismissed concerns by other members of the crew. Moreover the crew often failed to question or challenge the captain’s decisions ^2^. In medicine Atul Gawande (2014) ^3^ suggested that the behaviour of doctors medicalising old age and not accepting that life/death is not curable is a sign of hubris within the profession. He argues that doctors should shift away from simply fighting for longer life, for things that make life meaningful. A leading Medical School in UK decided that it is no longer enough to have high grades to become a medical student but would-be doctors must also display humility ^4^.

Phenomena of hubristic behavior were possibly present in investigators of at least some of the fraudster studies reported in recent years. Among numerous examples, three articles are cited here one from the physics world and two from medicine. Jan Hendrik Schön rose to prominence after a series of breakthroughs in semiconductors, most of them published in Nature and Science, which were later discovered to be fraudulent ^5^. Hwang-Woo-suk, until 2005, was considered one of the pioneering experts in the field of stem cells and was best known for two articles published in the journal *Science* in 2004 and 2005, where he reported that he had succeeded in creating human embryonic stem cells by cloning ^6^. He was called the "Pride of Korea" in South Korea. These reports were later found to be fabricated. Another tragic example of possible hubris was the report in Nature of a new and simple way to produce inducible stem cells. The method was soon found to be irreproducible and was retracted, but in the meantime, one of the senior authors committed suicide ^7^

The “intellectual celebrity syndrome” was implied by Winkler (1987) ^8^ for writers and scholars who risk seeking to popularise serious ideas or influence contemporary events by transmitting them to the general public in a distorted and unusable manner. Winkler suggested that this phenomenon resembles a disease characterised by the presence initially of a pleasant exhilaration, followed by celebration, eccentric indulgent and somnolent phantasies. Nobel Prize laureates who undertake projects or accept positions beyond their capabilities was described by Diamandis (2013) ^9^ as “Nobelitis”. Diamandis claims that the Nobel Prize seems to provide laureates with reassurance that they hold some super-powers that they did not realise before and that the prize will assist them to go on to even greater achievements.

The speed of communications in contemporary times, combined with the widespread application of social media and easy access to large groups of people, might predispose to both collective and individual hubristic decision making. The consequences of hubristic behaviour can be profound with dangerous consequences ^10^. Acclaimed scientists should exercise greater humility to the complexity and inherent uncertainty of their activities and strive to seek out and challenge hubristic behaviours. Mentors are encouraged to discuss hubris and related behaviours with their mentees and stress the importance of humility in their future activities.
