# Supplementary material for: Hubris and Sciences
Source: F1000Res. 2018 Feb 1;7:133. [Version 1] doi: 10.12688/f1000research.13848.1 (PMC5850088; doi:10.12688/f1000research.13848.1)
Supplement: Peer review history

# Peer review: Referee response for version 1

**DOI:** 10.5256/f1000research.15052.r30448

**Published:** 2018-03-12

**Authors:** Eugene Sadler-Smith

**Affiliations:** 1 Surrey Business School, University of Surrey, Guildford, UK

I enjoyed reading this interesting, relevant and well written article.

 I think that's it's great strength is the focus on hubris in scientific discovery - an area that is currently not written much about. I liked the examples and the arguments and I think they are convincing.

 If there are any areas for development maybe the authors could say a bit more about one of the really interesting points that they mention but don't develop -that is teaching humility, this is a really great and novel idea in hubris research and I like the idea of approaching it from a Socratic or Aristotelian perspective.

 Good luck I hope this work gets indexed.

I have read this submission. I believe that I have an appropriate level of expertise to confirm that it is of an acceptable scientific standard.

# Peer review: Referee response for version 1

**DOI:** 10.5256/f1000research.15052.r30450

**Published:** 2018-02-16

**Authors:** Eleni Palazidou

**Affiliations:** 1 Barts and The London School of Medicine and Dentistry, Queen Mary University of London (QMUL) , London, UK; 2 University of Nicosia Medical School, Nicosia, Cyprus

The ancient Greeks recognized the dangers of  “overvaluing” one’s self and hence coined the word Hubris (Ύβρις), meaning a highly exaggerated sense of self-importance that is an insult to the Gods.

 Anybody in position of power, in any sphere of life and the authors comment on scientists being afflicted by hubris as much as others. Very rightly they point out that scientific knowledge continuously grows and reinvents itself. In fact we can confidently (but not hubristically!) say, scientists will never manage to know everything! The growth of knowledge in every sphere is infinite.

 The authors comment on the “intellectual celebrity status” which has afflicted scientists. “Nobelitis” is mentioned as a syndrome of considering oneself as more able than one is, because of the high level of recognition afforded to them, which encourages hubristic behaviour.  Is prize giving, in general, feeding into hubris? It would have been good to expand on this.

 They recommend the cultivation of humility as a way forward and they urge mentors to teach the importance of humility and the dangers of hubristic behaviour. Can humility be taught and hubris prevented? The public’s thirst for celebrity status, which doesn’t spare the science world, works against this.

I have read this submission. I believe that I have an appropriate level of expertise to confirm that it is of an acceptable scientific standard.
